# Spatially controlled tenascin-C accumulation contributes to inflammatory disease persistence in giant cell aortitis

**DOI:** 10.1172/jci.insight.200255

**Published:** 2026-04-08

**Authors:** Hui Shi, Ying Tang, Jing Li, Ora Gewurz-Singer, Bo Yang, Dogukan Mizrak

**Affiliations:** 1 Department of Cardiac Surgery, University of Michigan, Ann Arbor, Michigan, USA.; 2 Department of Cardiovascular Surgery, Xiangya Hospital, and; 3 Department of Cardiology, Second Xiangya Hospital, Central South University, Changsha, China.; 4 Department of Vascular Surgery, Peking University People’s Hospital, Beijing.; 5 Department of Medicine, University of Michigan, Ann Arbor, Michigan, USA.

**Keywords:** Inflammation, Vascular biology, Cardiovascular disease, Molecular biology, Transcriptomics

## Abstract

Giant cell aortitis (GCA) is an inflammatory disease of the aortic wall with a characteristic giant cell pattern on pathology and can lead to life-threatening aortic aneurysm and dissection. Pathogenic GCA mechanisms underlying aortic inflammation and persistence remain elusive. Here, we demonstrate the complexity of medial layer destruction and immune cell infiltration in clinical granulomatous GCA and lymphoplasmacytic IgG4-related aortitis samples using imaging-based gene expression profiling. Single-cell spatial profiling revealed aortic wall remodeling in the GCA aortas, highlighting substantial phenotypic modulation in stromal cells, including vascular smooth muscle cells (SMCs) and fibroblasts. Specifically, we observed the expansion of stromal cells expressing Tenascin-C (*TNC*) mRNA and spatially refined TNC accumulation in lesion areas. We confirmed these findings histologically using diseased aortas resected from individuals with giant cell arteritis and clinically isolated aortitis. Mechanistically, our data suggest that TNC promotes a proinflammatory phenotype in primary human SMCs, elevating IL-6 levels partially through the TLR4/NF-κB pathway. IL-6 signaling propagates the proinflammatory loop by activating STAT3. Pharmacological blockade of the IL-6 receptor using tocilizumab alleviated the TNC-driven proinflammatory phenotype. We propose that TNC acts as a local catalyst of inflammatory disease persistence mainly via IL-6 signaling activation and offers a potential avenue for sustained disease remission.

## Abstract 2

Spatially Controlled Tenascin-C Accumulation Contributes to Inflammatory Disease Persistence in Giant Cell Aortitis

## Graphical abstract

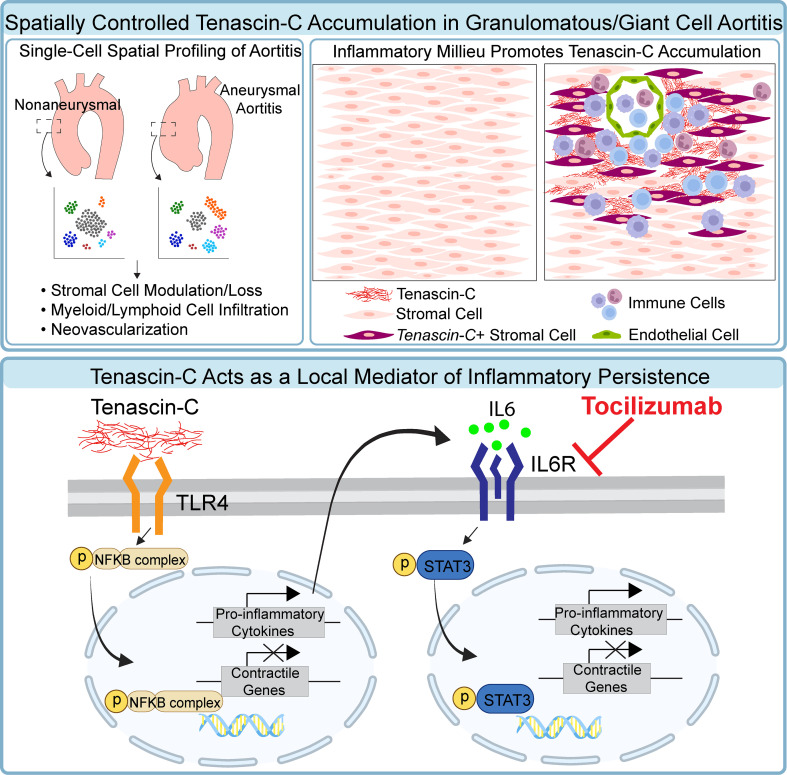

## Introduction

Aortitis is a comprehensive term describing excessive inflammation of the aortic wall, which can result in aortic aneurysm and dissection with high mortality rates (1, 2). Patients with aortitis might not present with obvious systemic vasculitis symptoms and the diagnosis is typically made postoperatively through histopathological evaluation of the resected aorta. Patients with surgically resected aortitis have high re-operation rates compared with aortic aneurysms without aortitis (3). In addition, these patients are at high risk of developing subsequent arterial lesions even after resection. With the recent advances in large-vessel imaging, including fluorodeoxyglucose–positron emission tomography, radiographic diagnosis of aortitis is becoming increasingly common (4, 5). Due to its challenging diagnosis, the prevalence of aortitis is currently unknown and likely underestimated. The histopathological evaluations of surgical samples estimated that 4% of thoracic aorta and up to 15% of ascending aorta repair patients have aortitis (6–9).

Aortitis can be broadly classified into noninfectious and infectious forms (10, 11). Noninfectious aortitis can be idiopathic, referred as clinically isolated aortitis (CIA) or secondary to systemic vasculitides and other inflammatory diseases, including giant cell arteritis, Takayasu arteritis, IgG4-related disease, and others (12, 13). Giant cell arteritis predominantly affects the temporal artery, causing cranial symptoms such as headache and jaw claudication; however, nearly 30% of patients with giant cell arteritis also develop large vessel complications primarily in the thoracic aorta (1, 14–16). High-dose glucocorticoids in conjunction with steroid-sparing immunosuppressant drugs are typically administered to manage giant cell arteritis with variable effectiveness and high relapse rates. Furthermore, prolonged glucocorticoid use has substantial toxicity and can lead to diabetes, coronary artery disease, and cataracts and increases the risk of severe infections. There is a need for more targeted, steroid-sparing therapeutic modalities.

There are several types of aortitis. Often presenting as a large vessel complication in association with giant cell arteritis, giant cell aortitis (GCA) is a relatively common aortitis type that shows a characteristic granulomatous giant cell pattern on pathology. Aortic complications can arise after the onset of giant cell arteritis or precede the cranial symptoms and initial diagnosis (12, 17). Histopathological evaluations revealed that CIA also shows granulomatous immune infiltrates, with a giant cell pattern in the immune-privileged aortic media, medial scarring, loss of vascular elasticity, and aortic wall thickening (18, 19). However, CIA is not affiliated with systemic disease. IgG4-related aortitis is characterized by lymphoplasmacytic infiltrate with a predominance of IgG4^+^ plasma cells. Notably, giant cell arteritis frequently affects female adults older than 50 years of age (12), while CIA can affect younger patients and show comparatively more male involvement (18, 19). In addition, patients with CIA can report systemic complications over time, suggesting some CIA cases may represent an early manifestation of underlying systemic vasculitides, such as giant cell arteritis (20, 21).

Recent studies on molecular mechanisms of aortitis evaluated levels of different immune cells and cytokines as potential biomarkers, and assessed the phenotypic changes in immune cells from myeloid and lymphoid lineages in noninfectious aortitis vessels (22). Among cytokines, IL-6 is implicated in inflammatory cascades, pathogenic T cell imbalance, as well as aortitis-associated fibrosis through JAK/STAT pathway activation (23, 24). IL-6 receptor (IL-6R) inhibition with an FDA-approved humanized monoclonal antibody against IL-6R, tocilizumab (TCZ), has been shown to improve clinical outcomes, becoming the gold standard first-line agent for the treatment of giant cell arteritis (24, 25). In the Giant Cell Arteritis Actemra (GiACTA) trial, although TCZ improved outcomes over steroids alone, not all patients achieved remission with this drug and relapse rates were high, demonstrating the need for additional therapeutic options (26).

Recent single-cell and single-nucleus RNA-sequencing studies began to identify the transcriptional mechanisms underlying human aortic diseases (27–30). Single-cell RNA sequencing of the human aorta comes with unique challenges, including cell dissociation difficulties due to the high extracellular matrix content of the vessel. We recently demonstrated the feasibility of high-resolution spatial profiling of the human aorta (31). This approach has not been utilized to study clinical human aortitis samples, which undergo spatial remodeling. Given the severity of aortitis, and limitations of the current targeted therapeutic modalities, detailed understanding of cellular mechanisms underlying disease pathology is of high importance. In this study, we investigated the aortitis tissue landscape using high-resolution spatial profiling of surgically resected clinical specimens and functionally characterized the role of Tenascin-C (TNC) in inflammatory disease persistence. Our findings suggest that interactions between the inflammatory milieu and tissue-resident cells play a vital role in orchestrating disease chronicity.

## Results

### Spatial landscape of vascular remodeling in aortitis vessels.

To investigate the aortic wall remodeling in aortitis samples at high resolution, we performed imaging-based spatial gene expression profiling on human ascending aortas collected from 3 female patients with aneurysm diagnosed with GCA showing presence of granulomatous inflammation with giant cells on pathology (denoted as GCA-1, GCA-2, and GCA-3), 2 female nonaneurysmal participants (denoted as Nonaneurysmal-1 and Nonaneurysmal-3), and 1 male nonaneurysmal participant (denoted as Nonaneurysmal-2) (Supplemental Table 1; supplemental material available online with this article; https://doi.org/10.1172/jci.insight.200255DS1). In addition, we performed the ascending aorta spatial profiling on 1 aneurysm patient diagnosed with IgG4-related aortitis (denoted as IgG4) with dense lymphoplasmacytic infiltrates and substantial IgG4^+^ plasma cell presence on pathology (Supplemental Table 1). The patients with aortitis had aneurysms of more than 5 cm in diameter in the ascending aorta and did not have a family history of aortic aneurysm or dissection (Supplemental Table 1). The patients with aortitis had either aortic insufficiency or no valvular complications, while the nonaneurysmal aortas were resected from patients with aortic stenosis (Supplemental Table 1). The spatial assays were performed using 10X Genomics Xenium Human Multi-tissue panel, which consists of 377 genes. This gene panel includes markers for different cell types, including vascular smooth muscle cells (SMCs), endothelial cells, and fibroblasts as well as diverse immune cells suitable to reveal the cellular complexity in aortitis samples. To confirm the cellular diversity in the GCA aorta, the spatial assay was conducted on 2 tissue sections isolated from neighboring aneurysmal regions (denoted as R1 and R2).

**Figure 1:**
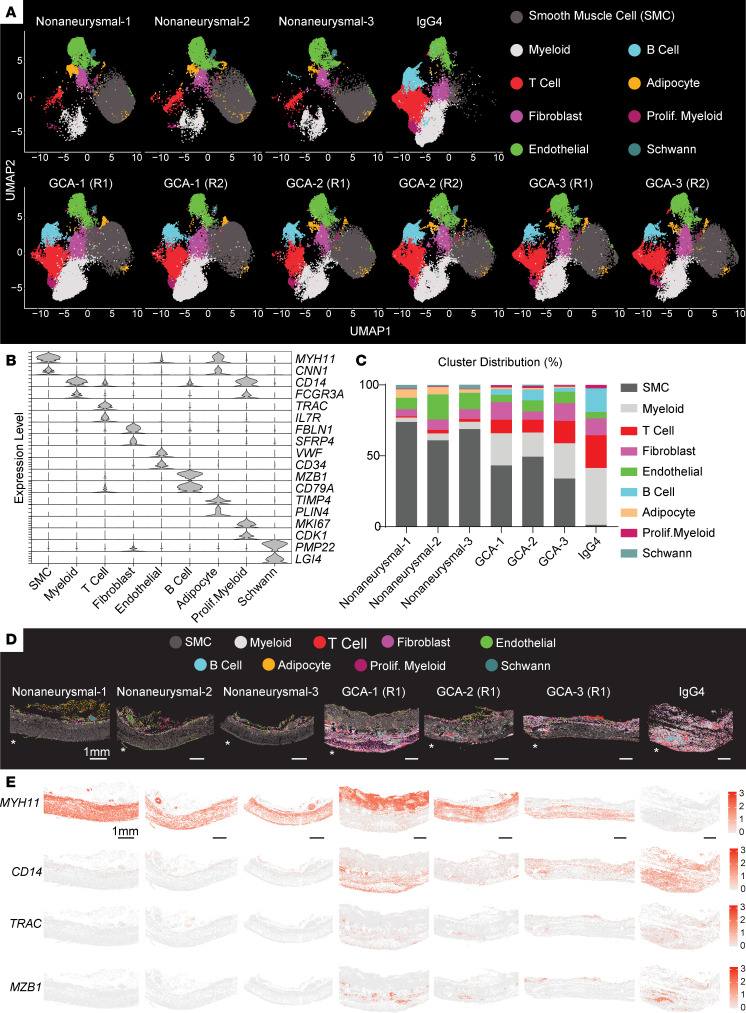
Spatial landscape of vascular remodeling in aortitis vessels. (**A**) Uniform Manifold Approximation and Projection (UMAP) visualization showing the sample distribution of 9 major clusters identified in 3 nonaneurysmal individuals, 1 IgG4-related aortitis sample, and in 3 patients with giant cell aortitis (GCA). The assay was performed in 2 independent regions (R1 and R2) per GCA patient. (**B**) Violin plots showing expression levels of significantly enriched markers across different cell clusters. (**C**) Cell cluster composition in each patient. Two sections were used to calculate cell composition for each GCA patient. The proliferating myeloid population is abbreviated as Prolif.Myeloid. (**D**) Spatial distribution of cell clusters across samples. Each circle represents an individual cell (centroid view) and is colored based on the cluster identification as in **A**. Asterisk (*) indicates the lumen. Uneven tissue edges introduced during tissue processing and loose perivascular tissue were trimmed to present the intact tissue segments in the spatial plots. Scale bars: 1 mm. (**E**) Spatially resolved normalized expression of smooth muscle and immune cell markers in each cell across different samples. Scale bars: 1 mm.

Unsupervised clustering revealed 9 major cell clusters with distinct marker expression: SMC cluster enriched for myosin heavy chain 11 (*MYH11*); myeloid cell cluster enriched for monocyte/macrophage markers (cluster of differentiation 14; *CD14* and Fcγ receptor IIIa; *FCGR3A*) (32); T lymphocyte cluster enriched for T cell receptor α constant (*TRAC*); fibroblast cluster enriched for fibulin 1 (*FBLN1*); endothelial cell cluster enriched for von Willebrand factor (*VWF*); B lymphocyte cluster enriched for marginal zone B– and B1 cell–specific protein (*MZB1*); perivascular adipocyte cluster enriched for adiponectin (*ADIPOQ*); a proliferating myeloid cell cluster enriched for both differentiated myeloid cell markers and cell proliferation markers such as marker of proliferation Ki-67 (*MKI67*); and Schwann cell cluster enriched for peripheral myelin protein 22 (*PMP22*) expression (Figure 1A, Supplemental Figure 1A, and Supplemental Dataset 1). The myeloid cell cluster was enriched for multiple myeloid lineage markers, including monocyte and granulocyte markers, suggesting additional heterogeneity (Supplemental Dataset 1). The major cluster markers exhibited pervasive and cluster-specific expression patterns except for the adipocyte cluster that likely contained SMCs showing their marker expression (Figure 1B). The cell clusters showed different distribution among different patient groups (Figure 1C). Relative SMC abundance diminished in the aortitis group, most apparently in the IgG4 aorta (Figure 1C). Immune cell clusters, including myeloid, T cell, and B cell clusters were more prevalent in the aortitis vessels, with myeloid cell cluster being the most abundant immune cell cluster (Figure 1C). Differential gene expression analysis confirmed the enrichment of immune markers and depletion of SMC markers in the aortitis samples (Supplemental Datasets 2 and 3). Next, we visualized the spatial distribution of major cell clusters in different samples (Figure 1D and Supplemental Figure 1B). As expected, the nonaneurysmal aorta walls were primarily comprised of SMCs, while the aortic media was comprised of immune cells and fibroblasts in the IgG4 aorta (Figure 1D). GCA aortas showed variable levels of medial damage, with the highest inflammation in GCA1 (Figure 1D). To better visualize the degree of inflammatory assault in the aortitis samples, we plotted the spatially resolved expression of different immune cluster markers (Figure 1E). *CD14*, *TRAC*, and *MZB1* expression was clearly visible in the aortic media in GCA1, GCA2, and IgG4 aortas, whereas they appeared more concentrated in the GCA3 adventitia, further confirming both myeloid and lymphoid lineage cell presence, including *MZB1^+^* B cells (Figure 1E).

### Subclustering analyses reveal proinflammatory vascular smooth muscle and fibroblast populations.

**Figure 2:**
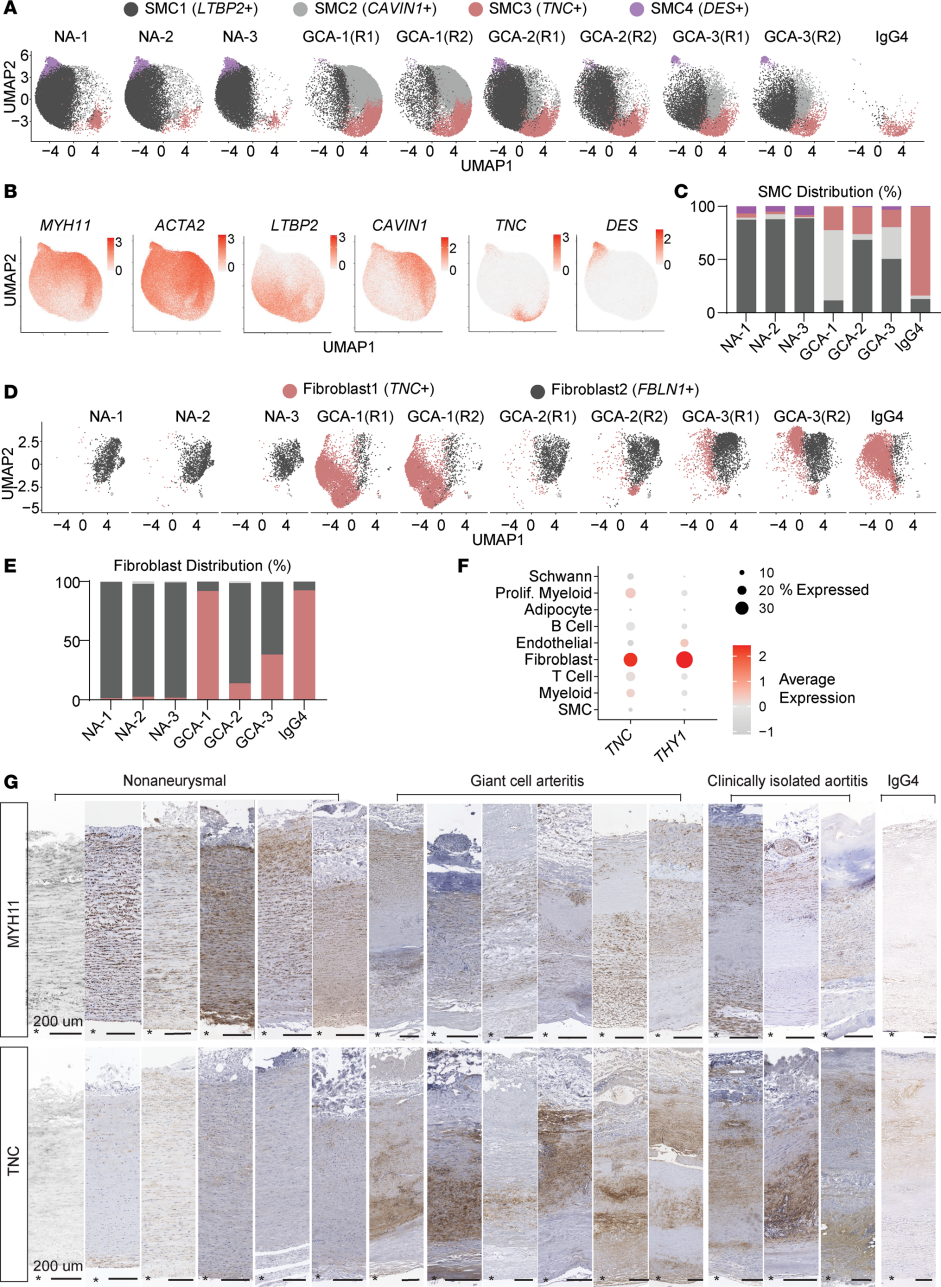
Subclustering analyses reveal proinflammatory vascular smooth muscle and fibroblast populations. (**A**) UMAP visualization of SMC subcluster sample distribution. NA, nonaneurysmal. (**B**) Normalized expression of SMC subcluster markers projected on merged UMAP visualization. (**C**) SMC subcluster composition in each participant. (**D**) UMAP visualization of Fibroblast subcluster sample distribution. (**E**) Fibroblast subcluster composition in each participant. (**F**) Dot plots showing *TNC* and *THY1* expression levels in each major cell cluster. (**G**) MYH11 and TNC immunohistochemistry in different samples. The detailed patient information is provided in Supplemental Table 2. Asterisk (*) indicates the lumen. Scale bars: 200 μm.

Spatial analysis of the major cell clusters in the aortitis vessels demonstrated a spectrum of inflammatory cell presence in the immune-privileged SMC-rich aortic media. We speculated that these immune infiltrates phenotypically modulate tissue-resident stromal cells, including SMCs and fibroblasts, which initiate defective repair mechanisms, compromising aortic structural integrity, ultimately leading to aneurysm formation. To resolve the molecularly distinct SMC subtypes, we performed subclustering analysis, which revealed 4 SMC subclusters, denoted as SMC1 to SMC4 (Figure 2A). The resulting subclusters showed distinct marker expression (Figure 2B and Supplemental Dataset 4). The contractile marker actin α2 (*ACTA2*) was more diffusively expressed, whereas *MYH11* appeared de-enriched in SMC3, suggesting a loss of SMC identity (Figure 2B). The SMC subclusters showed differential distribution among different patient groups (Figure 2C). Particularly, SMC1 enriched for latent TGF-β–binding protein 2 (*LTBP2*) and SMC4 enriched for desmin (*DES*) were more abundant in the nonaneurysmal aortas, while the aortitis samples were consistently more populated with SMC3 enriched for *TNC* (Figure 2C). Among the SMC subcluster markers, SMC4 marker *DES* showed a spatially distinct expression pattern labeling the SMCs at the media-adventitia junction (Supplemental Figure 1C).

Next, we subclustered fibroblasts, constituting another major cell population of the aorta. Subclustering revealed 2 major fibroblast subclusters (denoted as Fibroblast1 and Fibroblast2) and a small cell population constituting less than 1% of the fibroblasts (Supplemental Dataset 5). We observed the expansion of *TNC*^+^ Fibroblast1 in all aortitis vessels, with an enrichment in the GCA1 aorta (Figure 2, D and E). TNC is a large extracellular glycoprotein that participates in tissue inflammation and fibrosis in various organs (33–35). The *TNC*^+^ Fibroblast1 population also showed enrichment for aortic aneurysm–associated thrombospondin-2 (*THBS2*) (36, 37) and Thy-1 cell surface antigen (*THY1*) (Supplemental Dataset 5), which plays a context-dependent role in tissue fibrosis (38). Both GCA and IgG4 samples showed excessive collagen deposition in intima or adventitia, a common characteristic of fibrotic aortitis vessels (Supplemental Figure 1D) (1). Importantly, *TNC* and *THY1* were most enriched in the Fibroblast cluster among all major cell clusters, suggesting fibroblast phenotypic modulation in the aortitis samples (Figure 2F). However, *TNC* expression was not exclusive to the stromal cell populations showing expression in immune cells as well (Figure 2F).

We then performed immunohistochemistry for MYH11 and TNC on aortas collected from 6 clinically monitored patients with giant cell arteritis as well as ascending aortas collected from 6 nonaneurysmal participants to validate our findings (Supplemental Table 2). The nonaneurysmal participants in the validation stainings either had no valvular complications or presented with aortic insufficiency, better matching with the valvular phenotypes of the patients with GCA (Supplemental Table 2). In addition to the GCA and IgG4 aortas, we also examined granulomatous aortas collected from 3 clinically monitored male CIA samples to determine the pervasiveness of our findings. We included male nonaneurysmal participants in the validation cohort (Supplemental Table 2). MYH11 staining confirmed localized SMC loss in the GCA, CIA, and IgG4 aortas (Figure 2G and Supplemental Figure 2). TNC showed clear accumulation in the aortitis samples concentrating around lesion areas and was weak in the nonaneurysmal aortas (Figure 2G and Supplemental Figure 2). Taken together, these findings indicate substantial tissue remodeling and phenotypic modulation in stromal cells in the diseased aorta.

### TNC accumulates in aortitis lesions.

**Figure 3:**
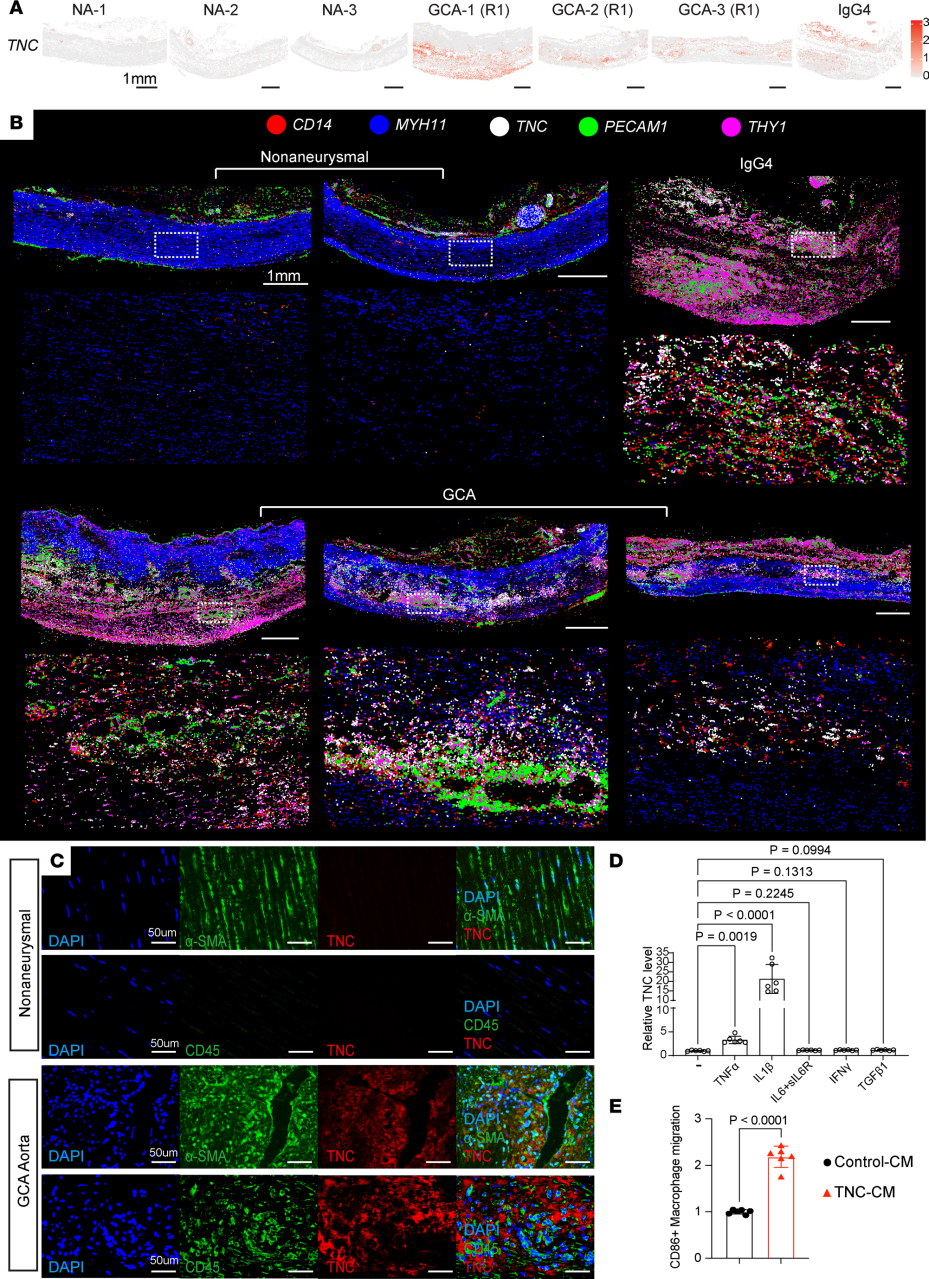
Tenascin-C accumulates in aortitis lesions. (**A**) Spatially resolved normalized *TNC* expression in each cell (centroid view) in different samples. NA, nonaneurysmal. Scale bars: 1 mm. (**B**) Spatial distribution of *CD14* (red), *MYH11* (blue), *TNC* (white), *PECAM1* (green), and *THY1* (magenta) transcripts in the aortic wall. Each enlarged circle represents an individual *CD14*, *MYH11*, *TNC*, *PECAM1*, or *THY1* transcript. Spatial distribution of molecules is plotted in **B**, while spatial distribution of cells is plotted in Figure 1D on the same sections. White dashed boxes indicate the magnified areas in each sample. Uneven tissue edges introduced during tissue processing and loose perivascular tissue were trimmed to present the intact tissue segments in the spatial plots. Asterisk (*) indicates the lumen. Scale bars: 1 mm. (**C**) Immunofluorescent staining for TNC, CD45, and α-SMA. Scale bars: 50 μm. (**D**) Relative *TNC* expression after the treatment of primary SMCs with various factors (*n* = 6, Kruskal-Wallis test with multiple comparisons). (**E**) CD86^+^ macrophage migration rate following control conditioned media (Control-CM) or TNC conditioned media (TNC-CM) treatment (*n* = 6, unpaired *t* test with Welch’s correction).

Next, we aimed to determine the molecular and cellular triggers for TNC expression in the GCA and IgG4 aortas. We first examined the spatially resolved expression of *TNC*, which predominantly labeled cells in the aortitis samples, albeit lacking a distinguishable spatially restricted expression pattern (Figure 3A). We then plotted individual molecule distribution for *TNC* and *THY1* along with SMC marker *MYH11*, endothelial marker *PECAM1*, and myeloid cell marker *CD14*. In the nonaneurysmal aortas, *MYH11*^+^ SMCs formed the aortic media, while *TNC* expression was negligible (Figure 3B). In both GCA and IgG4 aortas, the higher resolution images revealed accumulation of *TNC* molecules to aortic lesions concentrated around *CD14^+^* and *PECAM1^+^* areas (Figure 3B). The *PECAM1^+^* areas in the aortic media could indicate areas of neovascularization, which provides a gateway for immune cells to enter immune-privileged aortic media supporting chronic inflammation (39).

We then performed immunofluorescent staining and confirmed the expansion of TNC^+^α-SMA^+^ phenotypically modulated stromal cells in the GCA aorta (Figure 3C). We observed extracellular TNC accumulation in lesion areas populated with CD45^+^ immune cells (Figure 3C). As expected, TNC and pan-immune CD45 staining was nearly undetectable in the media of the nonaneurysmal aortas (Figure 3C). The accumulation of *TNC* molecules in areas of inflammation also implied molecular activation by inflammatory milieu. To inspect the molecular triggers of *TNC* expression, we stimulated primary SMCs with different proinflammatory and profibrotic cytokines. Recombinant TNF-α and IL-1β supplementation robustly activated *TNC* expression, while IL-6 and IFN-γ treatments had no effect (Figure 3D). In addition, profibrotic TGF-β1 treatment did not appear to induce *TNC* expression strongly (Figure 3D). To investigate the relevance of TNC accumulation to immune cell infiltration, we performed a Transwell migration assay on proinflammatory CD86^+^ human macrophages (Figure 3E). For robust TNC production, we transfected HEK-293 cells with a control or TNC-overexpressing (TNC-OE) plasmid and collected concentrated conditioned media from the HEK-293 cultures, denoted as Control-CM and TNC-CM, respectively (Supplemental Figure 3A). The cells were seeded in the top chamber and the lower chamber contained media supplemented with Control-CM or TNC-CM. TNC-CM induced CD86^+^ macrophage migration, suggesting a bidirectional interaction between inflammatory milieu and TNC regulation (Figure 3E). Overall, these data suggest that TNC accumulation in aortic lesions is regulated by proinflammatory factors such as TNF-α and IL-1β, which are primarily produced by immune cells, including myeloid lineage cells and lymphocytes (40, 41).

### TNC is a local catalyst of inflammation.

**Figure 4:**
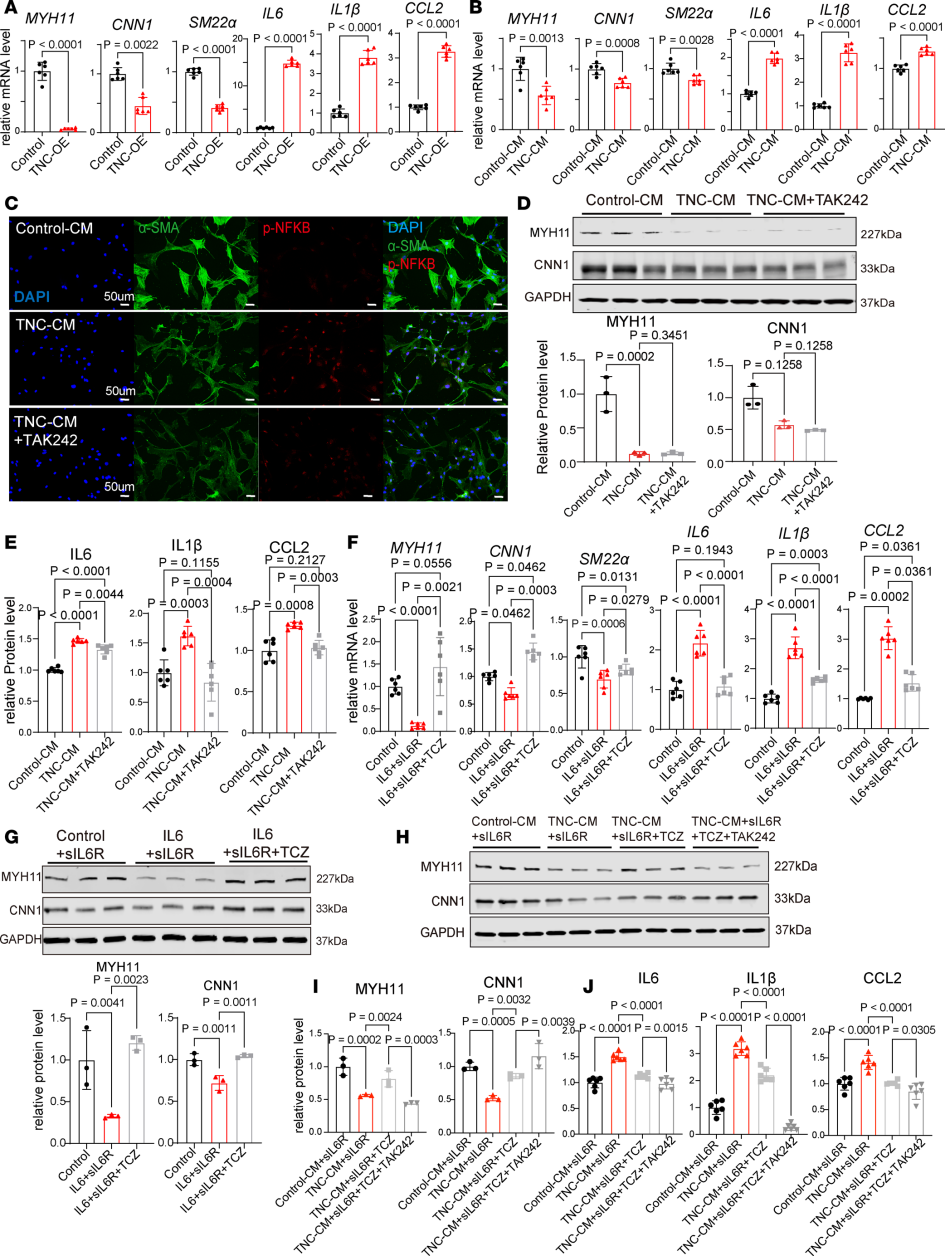
Tenascin-C is a local catalyst of inflammation. (**A**) Relative expression of SMC and inflammatory markers in primary SMCs overexpressing a control or *TNC* plasmid (*n* = 6, unpaired *t* test, unpaired *t* test with Welch’s correction, or Mann-Whitney test). (**B**) Relative expression of SMC and inflammatory markers in primary SMCs treated with Control-CM or TNC-CM (*n* = 6, unpaired *t* test or unpaired *t* test with Welch’s correction). (**C**) Immunofluorescence for p-NF-κB and α-SMA on primary cells after TNC-CM and TAK-242 (TLR4 inhibitor) treatments. Scale bars: 50 μm. (**D**) Western blots showing the levels of SMC markers after TNC-CM and TAK-242 treatments and their quantification (*n* = 3, 1-way ANOVA or Kruskal-Wallis test with multiple comparisons). (**E**) Quantification of IL-6, IL-1β, and CCL2 ELISAs on the conditioned media after Control-CM, TNC-CM, and TAK-242 treatments (*n* = 6, Brown-Forsythe and Welch’s ANOVA with multiple comparisons). (**F**) Relative expression of SMC and proinflammatory cytokines in primary SMCs treated with IL-6, soluble IL-6 receptor (sIL6R), and tocilizumab (TCZ) (*n* = 6, 1-way ANOVA, Brown-Forsythe and Welch’s ANOVA or Kruskal-Wallis test with multiple comparisons). (**G**) Quantification of MYH11 and CNN1 immunoblots after IL-6 signaling activation and blockade by TCZ (*n* = 3, 1-way ANOVA with multiple comparisons). (**H**) Western blots for MYH11 and CNN1 after Control-CM, TNC-CM, TCZ, and TAK-242 treatments. (**I**) Quantification of MYH11 and CNN1 Western blots (*n* = 3, 1-way ANOVA with multiple comparisons). (**J**) Quantification of IL-6, IL-1β, and CCL2 ELISAs in the conditioned media after Control-CM, TNC-CM, TCZ, and TAK-242 treatments (*n* = 6, 1-way ANOVA with multiple comparisons).

To assess the functional relevance of TNC, we first transfected primary human aortic SMCs with a TNC-OE or a control plasmid (Supplemental Figure 3B), and performed RT-qPCR 2 days later (Figure 4A). TNC-OE diminished the expression of SMC markers such as *MYH11*, calponin 1 (*CNN1*), and smooth muscle 22α (*SM22a*) while increasing *IL6*, *IL1b*, and C-C motif chemokine ligand 2 (*CCL2*) levels (Figure 4A). To stimulate the cells with extracellular TNC, we treated the primary SMCs with Control-CM or TNC-CM for 2 days, and performed RT-qPCR. TNC-CM significantly reduced SMC marker expression compared with Control-CM while inducing proinflammatory cytokine expression (Figure 4B). TNC signaling contributes to pathological tissue remodeling in various disease settings primarily via Toll-like receptor 4 (TLR4) (42, 43). TLR4 classically signals through the NF-κB complex to activate proinflammatory cytokine expression (44, 45). Immunofluorescent staining of primary SMCs showed increased NF-κB phosphorylation (p-NF-κB) and nuclear localization only 24 hours after TNC-CM treatment (Figure 4C and Supplemental Figure 3C). In the presence of a selective TLR4 inhibitor, TAK-242 (resatorvid) (46), TNC-CM or TNC-OE was unable to increase nuclear p-NF-κB levels, suggesting TNC action on NF-κB through TLR4 signaling (Figure 4C and Supplemental Figure 3, C and D). Interestingly, while immunoblots confirmed that TNC-CM reduces MYH11 and CNN1, TAK-242 was ineffective in restoring SMC marker levels (Figure 4D). To evaluate proinflammatory cytokine changes, we also performed enzyme-linked immunosorbent assays (ELISAs) on conditioned media collected from TNC-CM and Control-CM groups. ELISAs for IL-6, IL-1β and chemokine CCL2 showed their significant induction by TNC-CM (Figure 4E). TAK-242 reduced IL-1β and CCL2 to the baseline level, albeit only modestly suppressing IL-6 levels (Figure 4E).

IL-6 participates in the pathogenic cascade through STAT3 activation and IL-6 levels are elevated in patients with aortic aneurysms (47–50). Importantly, IL-6 signaling is indicated as a key target for vasculitis therapy (51, 52). Given the induction of IL-6 by TNC, we next evaluated the effects of IL-6 and TCZ on primary human SMCs. Treatment of cells with IL-6 and soluble IL-6R induced a proinflammatory phenotype with a significant reduction in SMC marker expression, while IL-6 signaling blockade with TCZ improved SMC marker expression (Figure 4, F and G). p-STAT3, but not p-NF-κB, markedly increased following IL-6 and soluble IL-6R treatments and p-STAT3 activation was suppressed by TCZ (Supplemental Figure 3E). To confirm that STAT3 is a driver of phenotypic changes in response to IL-6 signaling stimulation, we also treated the cells with 1 μM Stattic, a specific small-molecule inhibitor of STAT3 activation as confirmed by p-STAT3 immunoblots (Supplemental Figure 3, F and G) (53). Stattic diminished the effects of IL-6 activation on MYH11 and CNN1 levels in primary SMCs (Supplemental Figure 3, F and G). Notably, STAT3 is known to suppress contractile SMC marker expression by inhibiting the myocardin–serum response factor transcriptional complex (54) and is a transcriptional regulator of *IL6* expression in a positive feedback loop (55, 56), as also evidenced by the STAT3 binding peaks (ENCODE Project Consortium) (57) in the *IL6* promoter region (Supplemental Figure 3H).

Based on these results, we reasoned that the ineffectiveness of TAK-242 to restore SMC marker expression may be partially due to its weak IL-6 suppression. We next treated TNC-CM– and Control-CM–stimulated cells with TCZ. Immunoblots showed that TCZ treatment improved SMC marker levels in TNC-CM group (Figure 4H). CNN1 levels were improved in cells treated with both TCZ and TAK-242, while the combination treatment failed to rescue MYH11, a late-stage contractile SMC marker, suggesting their stage-specific effects (Figure 4, H and I). In addition, ELISAs showed that TCZ can suppress IL-6 and CCL2 levels, indicating the central role IL-6 signaling plays in aortic cell inflammation (Figure 4J). Treatment of the cells with both TCZ and TAK-242 also restored IL-1β levels showing potential benefits of using TAK-242 (Figure 4J). Collectively, our data imply that TNC broadly induces a proinflammatory loop in primary aortic SMCs and pharmacological blockade of IL-6 signaling is required to restore the contractile phenotype.

## Discussion

In this study, we investigated the complexity of aortic wall remodeling in aneurysmal aortitis using high-resolution spatial profiling of surgically resected samples. The GCA tissue landscape revealed substantial structural changes in immune-privileged aortic media, with myeloid and lymphocyte invasion, neovascularization-like formations, and widespread contractile SMC loss. Vascular inflammation is the key characteristic of aortitis; thus, synergistic interactions between T lymphocytes and myeloid cells that initiate the pathogenic process have been extensively studied (22, 58–64). We also detected variable amounts of B lymphocytes in the aortitis vessels with a clear enrichment in the lymphoplasmacytic IgG4 sample, confirming the different pathology of IgG4 compared with GCA. Therapeutic strategies aiming for B cells have shown promise in IgG4-related aortitis (65). B cell depletion with rituximab has been reported as a promising strategy to prevent IgG4-related disease relapse (66). In 2025, another monoclonal antibody targeting CD19 on B cells, Uplizna (inebilizumab-cdon), was FDA approved for use in IgG4 (67). Importantly, our data highlight that aortic stromal cells, including fibroblasts and SMCs, that are essential for vasular structural integrity acquire a proinflammatory phenotype orchestrated by immune infiltrates. We speculate that these dysfunctional stromal cells amplify vascular breach and potentiate inflammatory disease chronicity in the GCA aortas.

TNC accumulates in medial lesion areas in aortitis vessels. Our data indicate that TNF-α and IL-1β can robustly induce *TNC* expression, thereby likely mediating the TNC accumulation. Spatially controlled TNC aggregation in the aortitis samples localized near immune cells surrounding neovascularized regions particularly. Medial neovascularization in large vessel vasculitis can improve blood supply to hypoxic areas but also provides a cellular freeway for the ongoing inflammatory assault driving chronic inflammatory remodeling (68). Medial neovascularization also increases susceptibility to aortic dissection (39). TNC aggregation is observed at tissue injury sites in various pathological conditions, including autoimmune diseases and cancer (35, 69). The amount of TNC accumulation correlates with worse prognosis and higher cancer aggressiveness (70, 71). TNC buildup is detected at inflamed lesion areas in rheumatoid joints and TNC injection elevates joint inflammation in mice (42). TNC has also been implicated in vascular remodeling by promoting inflammation and thrombosis, ultimately causing neointimal hyperplasia in mice (72). In fact, TNC has been proposed as a central regulator of immune response that co-evolved with immunoglobulin-based adaptive immunity in vertebrates (73). Given the widespread relevance of TNC in inflammatory processes, we speculate that TNC accumulation may be relevant to other forms of inflammatory aortic aneurysms, including clinically isolated aortitis as confirmed by immunostaining.

Our functional data suggest that locally produced TNC elicits potent effects on proinflammatory cytokine expression and contributes to the persistence of the inflammatory environment in the aortitis vessels. While the selective TLR4 inhibitor TAK-242 was able to block NF-κB activation and partially suppress proinflammatory cytokine expression, it failed to restore SMC contractile marker and IL-6 levels, suggesting the activation of alternative signaling receptors by TNC. TNC is a large extracellular protein and contains multiple receptor binding sites (74). In addition to TLR4, TNC can serve as a ligand for the integrin family of cell adhesion receptors that are reported to promote the phenotypic modulation of vascular SMCs and fibroblasts (74–76). Among key alternative TNC targets, integrin α9β1 is implicated in the development of autoimmune diseases, including multiple sclerosis and rheumatoid arthritis (77). Blocking TLR4 using TAK-242 even reversed protective TCZ effects on MYH11. Interestingly, blocking integrin α9 signaling using an anti–integrin α9 antibody has been reported to elevate MYH11 levels (75).

Noninfectious aortitis is associated with high rates of aneurysm and patient mortality. Current medical therapies in managing noninfectious aortitis have limitations. Clinical evidence supports the adjunct use of TCZ with glucocorticoids to achieve better disease remission in giant cell arteritis (51, 78, 79). However, the disease relapse remains common due to persistence of vascular inflammation (80). Even after surgical resection of inflammatory aneurysms, up to 50% of patients will go on to develop another arterial event and might benefit from targeted medical management postoperatively. Our findings imply the contribution of dysfunctional stromal cells in exacerbating disease persistence and potential benefit of targeting local disease catalysts such as TNC. TNC promotes IL-6 expression in human cardiac myofibroblasts and in a mouse model of cardiac fibrosis, consistent with our data (81, 82). We found that pharmacological blockade of IL-6 signaling with TCZ is effective in improving both contractile gene and proinflammatory cytokine expression in TNC-CM–treated primary cells. However, it is difficult to reduce the complex inflammatory environment in clinical aortitis samples exclusively to IL-6 signaling, as TNC-producing phenotypically modulated stromal cells would reset the IL-6 and other proinflammatory pathways potentially causing disease relapse.

This study has several limitations. Aortitis is a very rare complication and demands long-term clinical monitoring for a comprehensive diagnosis, limiting our sample size. Importantly, a recent plasma proteomics study reported TNC enrichment in a large cohort of large vessel giant cell arteritis and Takayasu arteritis patients compared with healthy controls, suggesting the pervasiveness of our findings (83). Single-cell spatial transcriptomics requires freshly collected, high RNA quality samples, and nonaneurysmal heart transplant samples usually do not meet this criterion due to the relatively long wait times. Therefore, we collected fresh-frozen nonaneurysmal samples collected from valvulopathy patients in our single-cell spatial profiling. We performed histological validations on nonaneurysmal aortas collected from patients with aortic insufficiency or no valvulopathy to match the valvular features of the aortitis patients. Although we were unable to spatially profile the whole transcriptome, the Xenium gene panel included immune cell markers and inflammatory disease-associated genes and was sufficient to examine the cell diversity in the clinical aortitis samples. We used end-stage surgically resected aortitis vessels, which also have compensatory structural changes. Aortitis is a progressive disease, and we speculate that the ineffective compensatory changes initiate phenotypic modulation of stromal cells, which participate in the pathogenic process. Lastly, we utilized primary cells isolated from human aorta for functional assays as there is currently no universal in vivo model to study aortitis formation and progression. Primary aortic SMCs acquire fibroblast-like features after early passages but we demonstrate the inflammatory modulation of both stromal cell populations, SMCs and fibroblasts, in the aortitis samples, suggesting the relevance of the primary cell model. Despite these challenges, our findings indicate that interactions between immune and stromal cells cause dysfunctional vascular remodeling and drive spatially regulated TNC accumulation, which contributes to inflammatory disease persistence. TNC acts as a local disease catalyst notably via IL-6 signaling activation, providing a promising therapeutic target to achieve sustained GCA remission.

## Methods

### Sex as a biological variable.

Aortitis has a higher incidence among females (12). Therefore, we predominantly used samples collected from female participants in this study. Supplemental Tables 1 and 2 include detailed patient information.

### Spatial profiling of human aorta and data analysis.

Ascending aorta samples were surgically resected from nonaneurysmal participants with aortic stenosis and patients with aortitis (Supplemental Table 1). The tissue samples were isolated from the greater curvature of the ascending aorta. The fresh frozen aorta blocks were prepared as previously described (31). Briefly, the ascending aorta pieces were embedded and frozen in OCT compound. The RNA quality of each OCT block was determined using an Agilent Bioanalyzer. OCT blocks were cut into 10 μm sections in a cryostat and the sections were fixed in preheated paraformaldehyde. The tissue sections were then processed using the predesigned Human Multi-tissue and cancer panel (377 genes) using the Xenium in situ platform from 10X Genomics. We processed 2 tissue sections from neighboring ascending aortic regions for each patient with GCA (denoted as R1 and R2). Image decoding, cell segmentation, and transcript assignment were performed using the onboard Xenium analyzer, which uses DAPI expansion or multitissue staining for cell boundary estimates. Entire tissue pieces were used in the subsequent clustering and differential gene expression analysis. The Xenium output was inputted to Seurat 4.3 (R package) using LoadXenium function (84). Cells with less than 10 molecules were discarded from the subsequent analysis. The samples were normalized using and merged using SCTransform and merge functions, respectively. The merged dataset was scaled using ScaleData function. Unsupervised clustering (Louvain algorithm) and dimensionality reduction were done to identify spatially resolved cell clusters on the merged dataset. DimPlot, FeaturePlot, and ImageFeaturePlot functions were used to visualize the data on a 2-dimensional embedding. ImageDimPlot function was used to visualize the localization of individual molecules. Cluster markers were identified using PrepSCTFindMarkers and FindAllMarkers functions, respectively. Differentially expressed genes in different patient groups were determined using FindMarkers function, which uses Wilcoxon’s rank sum test with Bonferroni’s correction.

### Immunostainings and Masson’s trichrome staining.

Aortic tissue was fixed in 4% paraformaldehyde and embedded into paraffin blocks. Blocks were sectioned into 5-μm-thick slices and then underwent deparaffinization, rehydration, and heated-induced antigen retrieval. After completely cooling down, slides were incubated in hydrogen peroxide block solution (ab64264, Abcam), followed by protein block solution. Slides were incubated in the primary antibody overnight at 4°C. Then slides were incubated with biotinylated secondary antibody, then streptavidin peroxidase, DAB chromogen and substrate mixed solution. After Tris-buffered saline (TBS) washes, hematoxylin was applied to the slides, followed by differentiation and the bluing process. After dehydration, slides were mounted and imaged under a KEYENCE microscope (BZ-X810). For immunofluorescent staining, tissue sections were prepared as described above. Both tissue and monolayer culture cell slides were treated with 0.5% Triton X-100 for permeabilization and then 5% bovine serum albumin (BSA) for blocking. Subsequently, slides were applied with primary antibody in 1% BSA at 4°C overnight. After thorough washing, secondary antibodies (Jackson ImmunoResearch) were applied for 1 hour at room temperature. The monolayer cell culture slides were mounted in antifade mountant with DAPI (S36939, Invitrogen). The tissue slides were treated with VectorLabs TrueVIEW Autofluorescence Quenching solution for 45 seconds and incubated in DAPI for 5 minutes prior to mounting. All slides were imaged using a KEYENCE microscope (BZ-X810). For Masson’s trichrome staining, slides were incubated in Bouin’s Fluid (ab150686, Abcam) for 1 hour after deparaffinization and rehydration. Then they underwent working Weigert’s iron hematoxylin, Biebrich scarlet/acid fuchsin solution, phosphomolybdic/phosphotungstic acid solution, aniline blue solution, 1% acetic acid solution, dehydration and mounting, slides were imaged using the KEYENCE microscope. Supplemental Table 3 includes key resources used in the study.

### Primary cell culture experiments for functional assays.

Primary vascular SMCs were isolated as previously described (31, 85) from thoracic aorta of a female heart transplant donor. Primary SMCs were cultured in DMEM/F-12 (11-320-033, Gibco) containing 10% heat-inactivated fetal bovine serum (FBS) (A5256801, Gibco) and 1% penicillin-streptomycin (15140122, Gibco). Primary SMCs were transfected with either 0.5 μg/mL control plasmid (pRP[Exp]-EGFP-EF1A>ORF_Stuffer, Vectorbuilder) or TNC-OE plasmid (pRP[Exp]-EGFP-EF1A>hTNC, Vectorbuilder) using Lipofectamine 3000 (L3000001, Invitrogen). Twenty-four hours after the transfection, transfection efficiency was confirmed by GFP fluorescence and by measuring TNC mRNA and protein levels. DNase-digested mRNA and protein were extracted 2 days and 3 days after transfection, respectively. For TNC-CM treatment, HEK293T cells were transfected with either 0.5 μg/mL control or TNC-OE plasmid and CM were collected up to 4 days after transfection. The CM were concentrated using centrifugal filters (84-572A, Genesee Scientific). After 24 hours of serum deprivation (2% FBS), primary SMCs were treated with 5% control CM or TNC-CM. mRNA was extracted 2 days after treatments. We also treated primary SMCs with 15 μM TAK-242, performed immunofluorescent staining at 24 hours, and extracted protein 3 days after treatment. For the IL-6 experiments, we treated cells with 20 ng/mL recombinant IL-6 protein and 25 ng/mL soluble IL-6R with or without 50 μg/mL TCZ. mRNA and protein were extracted 2 and 3 days after treatments, respectively. For Stattic experiments, the cells were treated with 1 μM Stattic (Selleckchem) for 3 days, and protein was harvested. In the TNC-CM rescue experiments, the cells were treated with 50 μg/mL TCZ or 50 μg/mL TCZ with 10 μM TAK-242 for 3 days and protein was harvested. To test the effects of TNC-CM on CD86^+^ proinflammatory macrophages, THP-1 (88081201-1VL, Sigma-Aldrich) cells were first cultured in RPMI 1640 medium (11875093, Gibco) containing 10% heat-inactivated FBS (A5256801, Gibco) and 1% penicillin-streptomycin (15140122, Gibco). THP-1 cells were induced to naive macrophages using media with 100 ng/mL phorbol 12-myristate 13-acetate for 2 days and media without phorbol 12-myristate 13-acetate for 1 day. The cells were then treated with 50 ng/mL LPS and 20 ng/mL IFN-γ for 2 days to differentiate them into CD86^+^ proinflammatory macrophages. To measure migration rate, CD86^+^ macrophages were seeded onto the upper inserts of a Transwell assay kit (ECM 509, Sigma-Aldrich). After 24 hours of starvation, the cells were treated with 5% TNC-CM for 24 hours and migration rate was measured according to the manufacturer’s protocol.

### Quantitative real-time PCR.

Cell RNA was isolated using RNeasy Mini Kit (Qiagen) and reverse-transcribed into cDNA using the SuperScript III kit (18-080-051, Invitrogen). Quantitative real-time PCR with SYBR Green Supermix (172-5121, Bio-Rad) was performed using a Bio-Rad Real-Time PCR Detection System. Relative gene expression levels were normalized to *GAPDH* and quantified using the 2^–ΔΔCt^ method.

### Western blots and ELISA.

For Western blots, cells were lysed in radioimmunoprecipitation assay buffer (89901, Thermo Fisher Scientific) supplemented with protease inhibitor cocktail (11873580001, Roche) and PhosSTOP phosphatase inhibitors (4906845001, Roche). Proteins (15–20 μg each) were resolved in SDS-PAGE gels and then transferred onto nitrocellulose membranes. Membranes were blocked with 5% nonfat milk (1706404XTU, Bio-Rad) and then incubated in primary antibody overnight at 4°C. After 3 washes, membranes were incubated with secondary antibodies (Li-Cor Biosciences) and scanned with the LI-COR DLx Odyssey imaging system. IL-1β, IL-6, and CCL2 levels were measured using Human IL-1β/IL-1F2 DuoSet ELISA Kit (DY201, R&D Systems), Human IL-6 DuoSet ELISA Kit (DY206, R&D Systems), and Human CCL2/MCP-1 DuoSet ELISA Kit (DY279, R&D Systems) according to the manufacturer’s protocols. The concentrations of inflammatory factors in both control and treatment groups were calculated after baseline Control-CM and TNC-CM normalization.

### Statistics.

When analyzing 2 datasets that were normally distributed (Shapiro-Wilk normality test), we performed 2-tailed unpaired *t* test. If the SDs were significantly different, we performed an unpaired *t* test with Welch’s correction. When analyzing 2 datasets that were not normally distributed, we used a 2-tailed Mann-Whitney test. When analyzing more than 2 groups that were normally distributed, we performed 1-way ANOVA with the 2-stage step-up method of Benjamini, Krieger, and Yekutieli for multiple comparisons. If the SDs were significantly different, we performed Brown-Forsythe and Welch’s ANOVA tests with the 2-stage step-up method of Benjamini, Krieger, and Yekutieli for multiple comparisons. When analyzing more than 2 groups that were not normally distributed, we performed a Kruskal-Wallis test with the 2-stage step-up method of Benjamini, Krieger, and Yekutieli for multiple comparisons. Individual *P* values are presented in the figures up to the significance level of *P* less than 0.0001. A *P* value of less than 0.05 was considered statistically significant. The data are presented as mean ± SD and the statistical analyses were performed using GraphPad Prism 10 software.

### Study approval.

Human participant studies were performed according to the protocols approved by the Institutional Review Board at the University of Michigan (HUM00054585, HUM00096079, and HUM00052866).

### Data availability.

Spatial gene expression profiling data have been deposited to the NCBI Gene Expression Omnibus under the accession number GSE306150. Quantitative data points in figure panels are reported in the Supporting Data Values file.

## Author contributions

HS, YT, DM, and JL performed the experiments and analyzed the data. DM and BY conceived the study and supervised the project. DM, OGS, HS, and BY wrote the manuscript.

## Funding support

This work is also in part the result of NIH funding and is subject to the NIH Public Access Policy. Through acceptance of this federal funding, the NIH has been given a right to make the work publicly available in PubMed Central.

- University of Michigan Frankel Cardiovascular Center MI-AORTA Aortitis Grant (to DM).
- NIH grant R01HL151776 (to BY).
- NIH grant R01HL176683 (to DM).

## Supplementary Material

Supplemental data

Supplemental data set 1

Unedited blot and gel images

Supporting data values

## Version 1 04/08/2026

Electronic publication
