# Supplementary material for: Spatially controlled tenascin-C accumulation contributes to inflammatory disease persistence in giant cell aortitis
Source: JCI Insight. 2026 Apr 8;11(7):e200255. doi: 10.1172/jci.insight.200255 (PMC13134725; doi:10.1172/jci.insight.200255)
Supplement: Supplemental data [file jciinsight-11-200255-s229.pdf]

SUPPLEMENTAL MATERIAL

Spatially Controlled Tenascin-C Accumulation Contributes to Inflammatory Disease Persistence in Giant Cell Aortitis

Hui Shi[1,2,+]; Ying Tang[1,3,+]; Jing Li[1,4]; Ora Gewurz-Singer[5]; Bo Yang[1,*]; Dogukan Mizrak[1,*]

[1]Department of Cardiac Surgery, University of Michigan, Ann Arbor, MI, USA; [2]Department of Cardiovascular Surgery, Xiangya Hospital, Central South University, Changsha, China; [3]Department of Cardiology, Second Xiangya Hospital, Central South University, Changsha, China; [4]Department of Vascular Surgery, Peking University People's Hospital, Beijing; [5]Department of Medicine, University of Michigan, Ann Arbor, MI, USA.

[+]These authors contributed equally to this work.

*Addresses for Correspondence:
Dogukan Mizrak, 2800 Plymouth Road, NCRC-26, Room 263S, Ann Arbor, MI, 48109. Email: dmizrak@med.umich.edu
Bo Yang, 1500 E Medical Center Dr. 5144 Frankel Cardiovascular Center, Ann Arbor, MI, 48109. Email: boya@med.umich.edu

Supplemental Material:

Supplemental Figures 1-3

Supplemental Tables 1-3

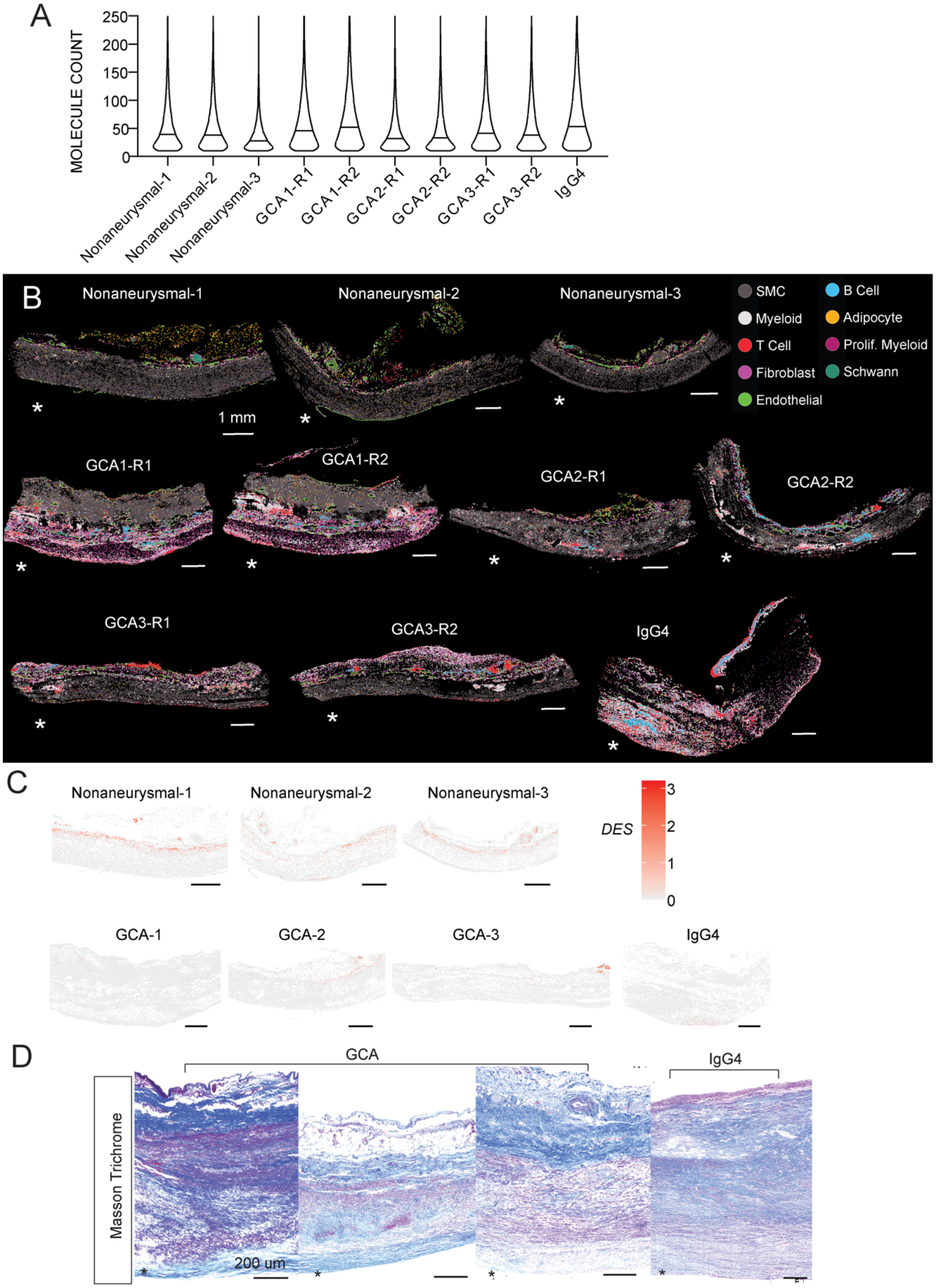

Supplemental Figure 1. A) Violin plots showing molecule counts per cell in each sample. Horizontal line indicates the median cell molecule count in each sample. **B)** Spatial distribution of cell clusters in each sample using untrimmed full images (Related

to Figure 1D). **C)** Spatially resolved *desmin* (*DES*) expression in each sample. **D)** Masson Trichrome staining showing collagen deposition in aortitis samples. Asterisk (*) indicates the lumen side. GCA: Giant cell aortitis; IgG4: IgG4 related aortitis. Scale bars: 200 μm.

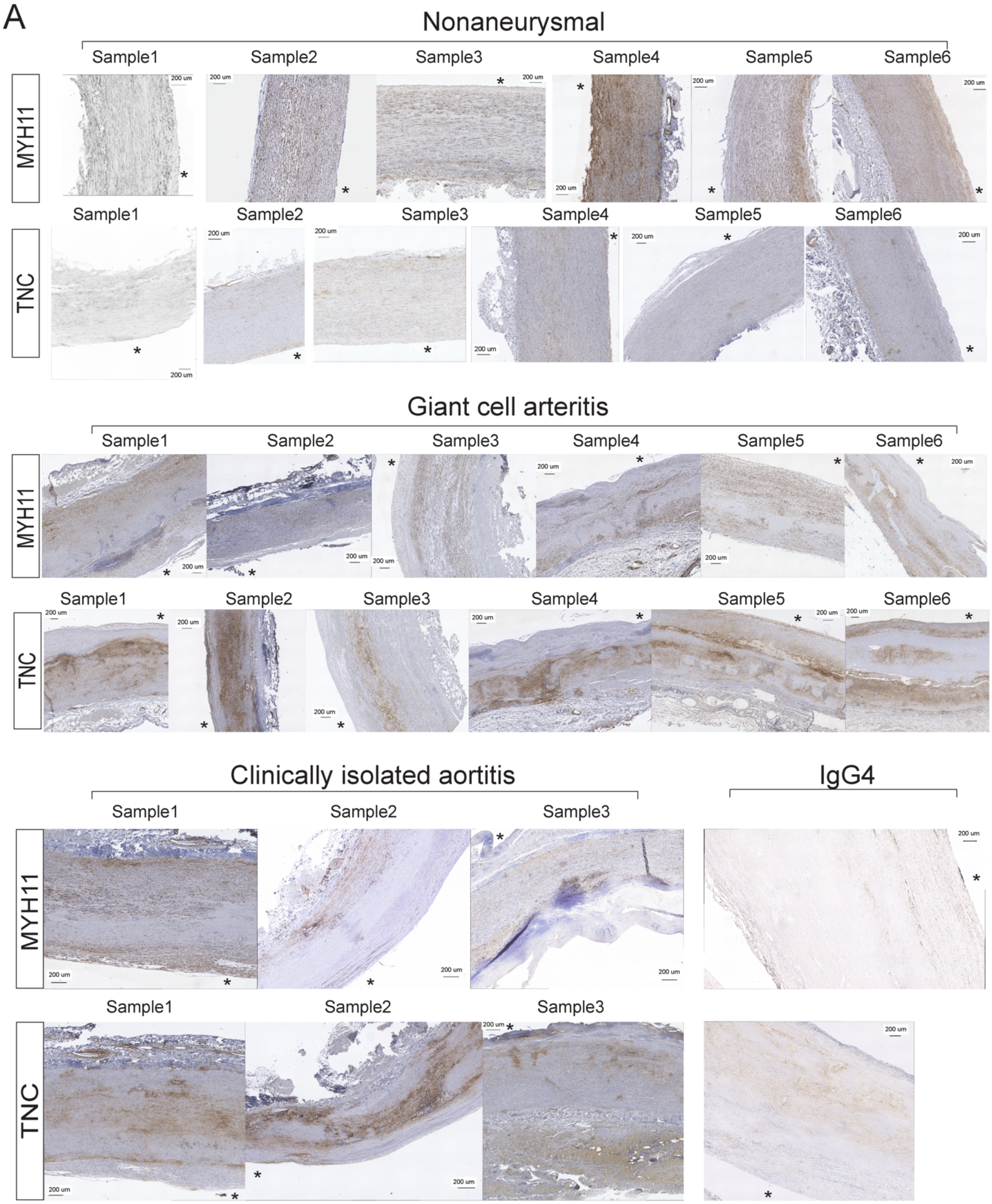

Supplemental Figure 2. Immunohistochemistry for MYH11 and TNC. The images correspond to the cropped images in Figure 2G. The patient information is provided in Supplemental Table 2. Asterisk (*) indicates the lumen side.

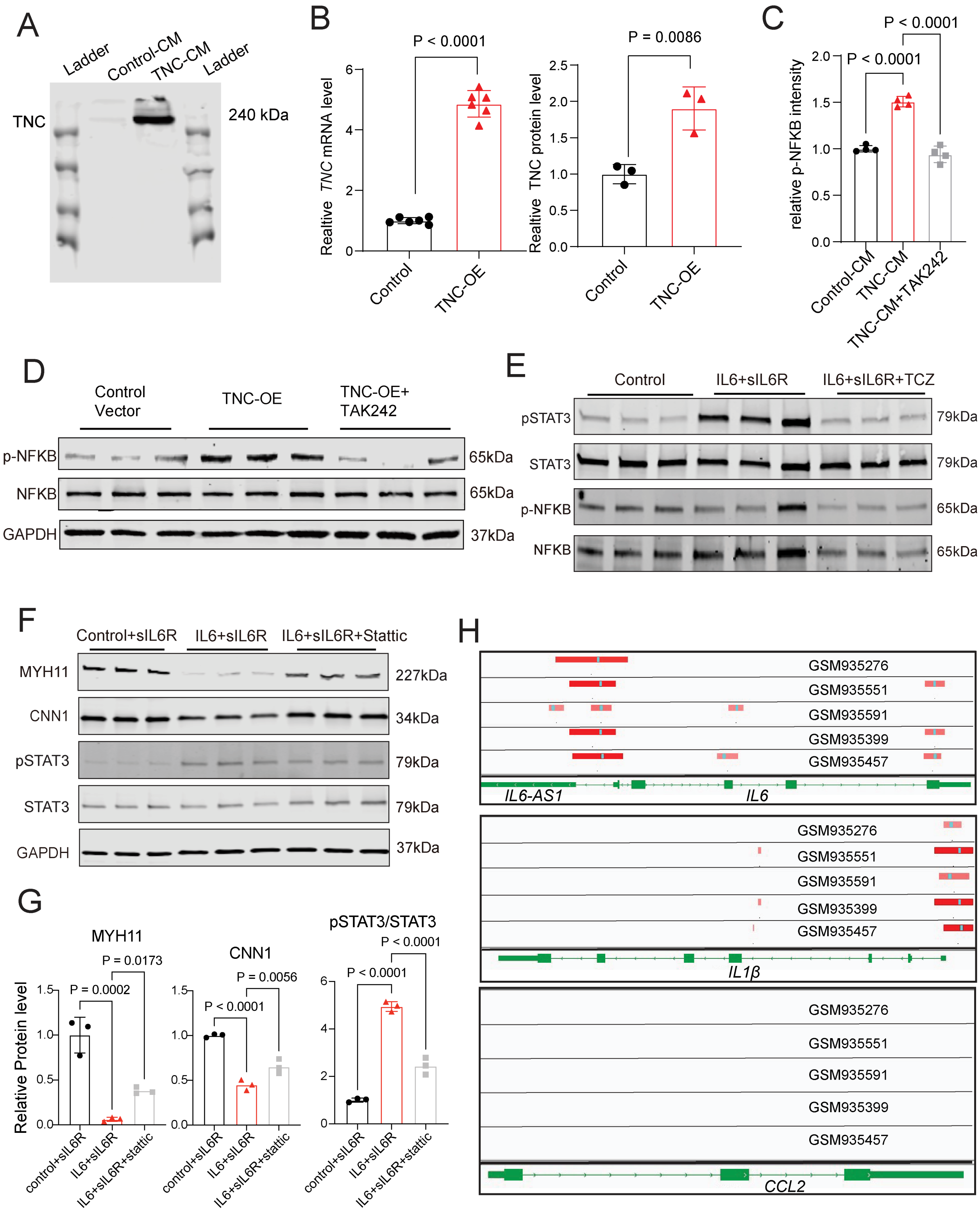

Supplemental Figure 3. A) Western blot for Tenascin-C in Control-CM and TNC-CM collected from HEK-293 cultures after a control or *TNC* plasmid over-expression. **B)** Quantification of RT-qPCR (unpaired t test with Welch's correction) and Western blot (unpaired t test) for Tenascin-C in primary smooth muscle cells overexpressing *TNC* (TNC-OE)**. C)** Quantification of nuclear p-NFKB staining intensity after TNC-CM and TAK-242 treatment (n=4, one-way ANOVA with multiple comparisons). **D)** Western blots on cell lysates for p-NFKB and NFKB after *TNC* over-expression (TNC-OE) and TAK-242 supplementation. **E)** Western blots on cell lysates for p-STAT3 and STAT3 after IL6, soluble IL6 receptor (sIL6R) and Tocilizumab (TCZ) supplementation. **F)** Western blots for MYH11, CNN1, p-STAT3 and STAT3 after IL6, soluble IL6 receptor (sIL6R) and 1 μM Stattic treatment. **G)** Quantification of MYH11, CNN1, p-STAT3 and STAT3 western blots after IL6, sIL6R and 1 μM Stattic treatment (n=3, one-way ANOVA with multiple comparisons). **H)** Integrative Genomics Viewer visualization of STAT3 CHIP-seq peaks mapping to *IL6, IL1β* and *CCL2.* Binding sites of STAT3 were obtained from ENCODE Project Consortium CHIP-seq data in Gene Expression Omnibus database GSE31477.

SAMPLE ID	AGE	SEX	ANEURYSM TYPE	ANEURYSM SIZE	VALVULOPATHY	CLINICAL FINDINGS
Nonaneurysmal-1	62	F	NA	NA	AS and Mild AI	Aortic stenosis, Hypertension, Dyslipidemia
Nonaneurysmal-2	59	M	NA	NA	AS and Moderate AI	Aortic stenosis, Hypertension, Dyslipidemia
Nonaneurysmal-3	65	F	NA	NA	AS and Mild AI	Aortic stenosis
GCA-1	57	F	Ascending	5.7 cm	Moderate AI	Giant cell aortitis, Aortic aneurysm, Pulmonary nodules, Hypertension, Muscle pain, Osteoarthritis
GCA-2	83	F	Ascending	5.5 cm	None	Giant cell aortitis, Aortic aneurysm, Hypertension, Dyslipidemia, Optic neuritis
GCA-3	76	F	Ascending	5.5 cm	Moderate AI	Giant cell aortitis, Aortic aneurysm, Hypertension, Dyslipidemia, Diplopia, Myopia, Presbyopia, Family history of neck/brain aneurysm
IgG4	66	F	Ascending	5.6 cm	AI	IgG4 related aortitis, Aortic aneurysm, Dyslipidemia, Cervicalgia, Glaucoma

Supplemental Table 1. The information about the samples used in spatial profiling. None of the patients were tobacco users at the time of surgery. The patients presented with tricuspid aortic valve except for Nonaneurysmal-2 who presented with bicuspid aortic valve. The patients did not have a family history of aortic aneurysm and dissection. GCA: Giant cell aortitis, IgG4: IgG4 related aortitis. F: female, M: Male, AS: Aortic stenosis, AI: Aortic insufficiency.

SAMPLE ID	AGE	SEX	ANEURYSM TYPE	ANEURYSM SIZE	VALVULOPATHY	CLINICAL FINDINGS
Nonaneurysmal-Sample1	59	F	NA	NA	AI	Aortic Insufficiency
Nonaneurysmal-Sample2	58	F	NA	NA	None	Heart Transplant, Hypertension
Nonaneurysmal-Sample3	58	F	NA	NA	None	Heart Transplant, Hypertension, Dyslipidemia
Nonaneurysmal-Sample4	37	M	NA	NA	None	Heart Transplant, Hypertension
Nonaneurysmal-Sample5	66	M	NA	NA	None	Heart Transplant, Hypertension
Nonaneurysmal-Sample6	56	M	NA	NA	None	Heart Transplant, Hypertension, Dyslipidemia
Giant cell arteritis-Sample1	75	M	Ascending	5.6 cm	None	Giant cell arteritis, Aortic aneurysm, Hypertension, Dyslipidemia
Giant cell arteritis-Sample2 (GCA-2 in Supplemental Table 1)	83	F	Ascending	5.5 cm	None	Giant cell arteritis, Aortic aneurysm, Hypertension, Dyslipidemia, Optic neuritis
Giant cell arteritis-Sample3 (GCA-3 in Supplemental Table 1)	76	F	Ascending	5.5 cm	Moderate AI	Giant cell arteritis, Aortic aneurysm, Hypertension, Dyslipidemia, Diplopia, Myopia, Presbyopia, Family history of neck/brain aneurysm
Giant cell arteritis-Sample4	69	F	Ascending	5.4 cm	AI	Giant cell arteritis, Aortic aneurysm, Hypertension, Dyslipidemia, Family history of abdominal aortic aneurysm
Giant cell arteritis-Sample5	64	F	Ascending	5.2 cm	None	Giant cell arteritis, Aortic aneurysm, Hypertension, Dyslipidemia
Giant cell arteritis-Sample6	67	F	Ascending	4.9 cm	None	Giant cell arteritis, Aortic aneurysm
Clinically isolated aortitis-Sample1	65	M	Ascending, Root	5.3 cm	AI	Clinically isolated aortitis, Aortic aneurysm, Hypertension, Dyslipidemia
Clinically isolated aortitis-Sample2	82	M	Ascending, Arch	5.7 cm	AS and AI	Clinically isolated aortitis, Aortic aneurysm, Hypertension, Dyslipidemia
Clinically isolated aortitis-Sample3	68	M	Ascending	5.5 cm	None	Clinically isolated aortitis, Aortic aneurysm, Hypertension, Dyslipidemia
IgG4-Sample1 (IgG4 in Supplemental Table 1)	66	F	Ascending	5.6 cm	AI	IgG4 related aortitis, Aortic aneurysm, Vasculitis, Dyslipidemia, Cervicalgia, Glaucoma

Supplemental Table 2. The information about the samples used in histological validations. The patients did not have a significant family history of aortic aneurysm or dissection except for giant cell arteritis-Sample4. F: female, M: Male, AS: Aortic stenosis, AI: Aortic insufficiency.

**Primers**	Sequence
GAPDH	F1: CAGCCTCAAGATCATCAGCA<br>R1: TGTGGTCATGAGTCCTTCCA
TNC	F1: CCTTGCTGTAGAGGTCGTCA<br>R1: CCAACCTCAGACACGGCTA
TAGLN (SM22α)	F1: AACAGCCTGTACCCTGATGG<br>R1: TGTCAGTCTTGATGACCCCA
MYH11	F1: CTTGGAAGCCAAAGAGGAAC<br>R1: CATCTCCTCCATCTGGGTCT
CNN1	F1: GTCCACCCTCCTGGCTTT<br>R1: AAACTTGTTGGTGCCCATCT
IL1β	F1: AGCTACGAATCTCCGACCAC<br>R1: CGTTATCCCATGTGTCGAAGAA
IL6	F1: ACTCACCTCTTCAGAACGAATTG<br>R1: CCATCTTTGGAAGGTTCAGGTTG
CCL2	F1: GATCTCAGTGCAGAGGCTCG<br>R1: TTTGCTTGTCCAGGTGGTCC

**Antibodies**		
TNC antibody	Cell Signaling	Cat#33352; RRID: AB_3096005
MYH11 antibody	Abcam	Cat# ab133567; RRID: AB_2890982;
GAPDH antibody	Cell Signaling	Cat#5174; RRID: AB_10622025
CNN1 antibody	Sigma	Cat#C2687; RRID: AB_476840
SM22α antibody	Abcam	Cat#ab14106; RRID: AB_443021
GAPDH antibody	Cell Signaling	Cat#97166; RRID: AB_2756824
NFKB antibody	Cell Signaling	Cat#8242; RRID: AB_10859369
p-NFKB antibody	Cell Signaling	Cat#3033; RRID: AB_331284
STAT3 antibody	Cell Signaling	Cat#9139; RRID: AB_331757
pSTAT3 antibody	Cell Signaling	Cat#9145; RRID: AB_2491009
CD45 antibody	Proteintech	Cat# 20103-1-AP; RRID: AB_2716813
α-SMA antibody	Sigma	Cat#A5228; RRID: AB_262054

**Chemicals, peptides, and recombinant proteins**		
Dulbecco's Modified Eagle Medium/F12 (DMEM/F12)	Gibco	11320-033
RPMI 1640 Medium	Gibco	11875093
Fetal bovine serum (FBS)	Gibco	A52567-01
Penicillin-Streptomycin	Gibco	15140-122
DPBS	Gibco	14190144

0.25% Trypsin-EDTA	Gibco	25200-056
Opti-MEM	Gibco	31985-070
Human IL-1 beta Recombinant Protein	PeproTech	200-01B
Recombinant Human soluble IL-6R alpha Protein	R&D Systems	227-SR
Recombinant Human TNF-alpha Protein	R&D Systems	210-TA
Human IFN-gamma Recombinant Protein	PeproTech	300-02
Human TGFβ1	PeproTech	100-21C
LPS	Sigma-Aldrich	L2880-10MG
TAK-242	Cayman chemical	13871
Tocilizumab	Selleckchem	A2012
Phorbol 12-myristate 13-acetate (PMA)	Sigma-Aldrich	79346
Control plasmid	VectorBuilder	pRP[Exp]-EGFP-EF1A>ORF_Stuffer
Tenascin-C plasmid	VectorBuilder	pRP[Exp]-EGFP-EF1A>hTNC
Paraformaldehyde solution	ChemCruz	sc-281692
Xylene	Fisher Scientific	C8H10
Citrate Buffer	Sigma-Aldrich	C9999
TBS Buffer	Thermos scientific	28358
Hematoxylin Gill	Fisher Scientific	245-654
Sodium Bicarbonate	Fisher Scientific	S233-2
Hydrochloric acid	Sigma-Aldrich	320331
Bovine Serum Albumin	Sigma-Aldrich	A7906
Triton X-100	Electrophoresis	BP151-100
ProLong Gold antifade reagent with DAPI	Invitrogen	P36935
**Commercial assays**		
Trichrome Stain Kit	Abcam	AB150686
Mouse and Rabbit Specific HRP/DAB Detection IHC kit	Abcam	AB64264
Lipofectamine 3000 Transfection Kit	Invitrogen	L3000015
SuperScript Ⅲ First-Strand	Invitrogen	18080051

Human Tenascin-C ELISA Kit (TNC)	Abcam	AB213831
Human IL-1 beta/IL-1F2 DuoSet ELISA Kit	R&D systems	DY201
Human IL-6 DuoSet ELISA Kit	R&D systems	DY206
Human CCL2/MCP-1 DuoSet ELISA Kit	R&D systems	DY279
RNeasy Mini Kit	Qiagen	74106
EndoFree Plasmid Maxi Kit	Qiagen	12362
TrueVIEW Autofluorescence Quenching	Vector Laboratories	SP-8400-15
QCM Chemotaxis Cell Migration Assay, 24-well (8 μm), fluorimetric	Sigma-Aldrich	ECM509
**Software and algorithms**		
ImageJ	Fiji	https://imagej.net/ij/
GraphPad Prism 10.1.2	GraphPad	https://www.graphpad.com/
Image Studio 6.0	LICORbio	https://www.licorbio.com/image-studio
LI-COR Acquisition Software	LICORbio	https://www.licorbio.com/las
BZ-X800 Viewer	KEYENCE	https://www.keyence.com/products/microscope/fluorescence-microscope/
Bio-Rad CFX Maestro	Biorad	https://www.bio-rad.com/en-us/product/cfx-maestro-software-for-cfx-real-time-pcr-instruments?ID=OKZP7E15
Xenium Analyzer	10X Genomics	https://www.10xgenomics.com/products/xenium-analysis
Seurat 4.3	R package	https://satijalab.org/seurat/

Supplemental Table 3. Key Resources.
