# Supplementary material for: Spatially controlled tenascin-C accumulation contributes to inflammatory disease persistence in giant cell aortitis
Source: JCI Insight. 2026 Apr 8;11(7):e200255. doi: 10.1172/jci.insight.200255 (PMC13134725; doi:10.1172/jci.insight.200255)

Full unedited blot for Figure 4D

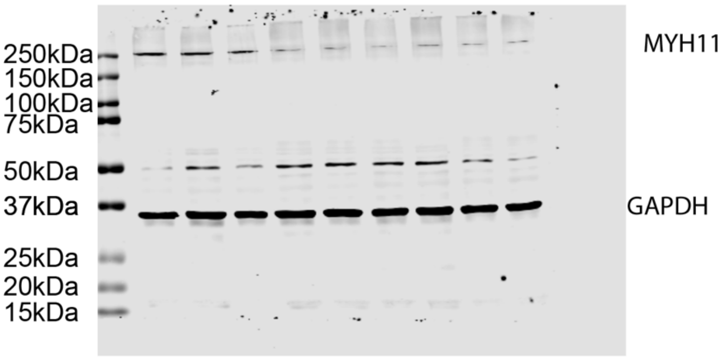


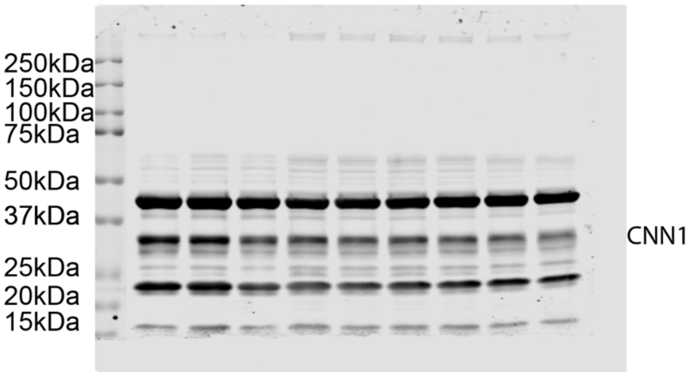

Full unedited blot for Figure 4G

MYH11

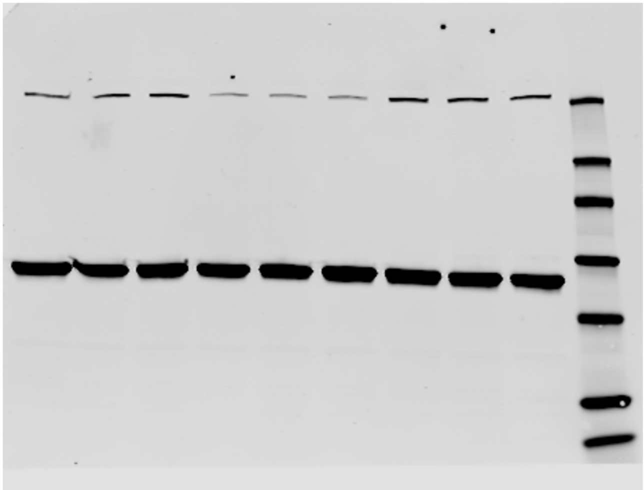

250kDa
130kDa
100kDa
70kDa
55kDa
35kDa
25kDa
15kDa

GAPDH

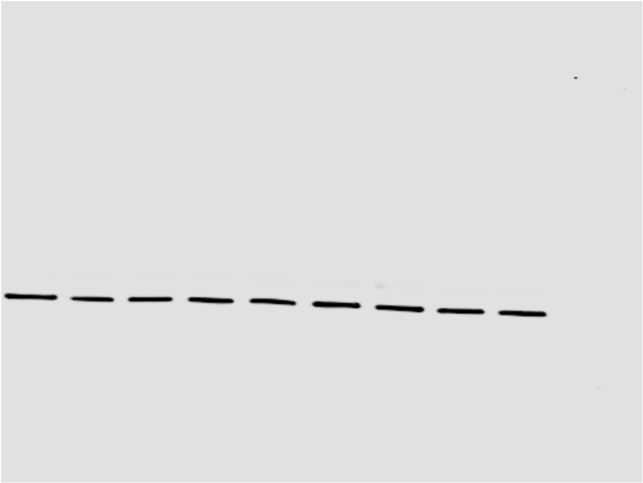

CNN1

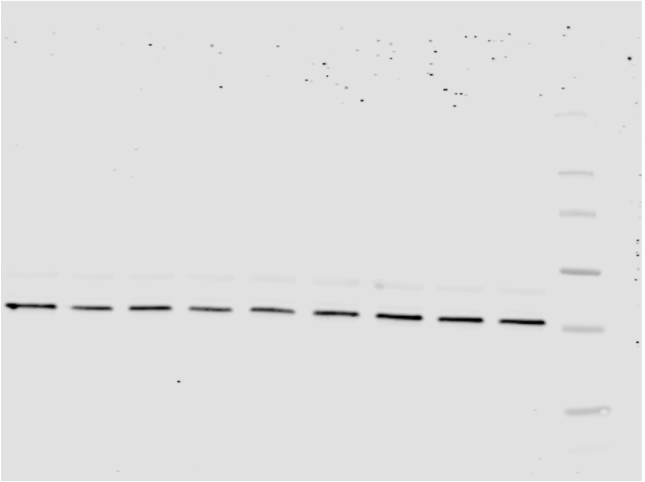

250kDa
130kDa
100kDa
70kDa
55kDa
35kDa
25kDa
15kDa

Full unedited blot for Figure 4H

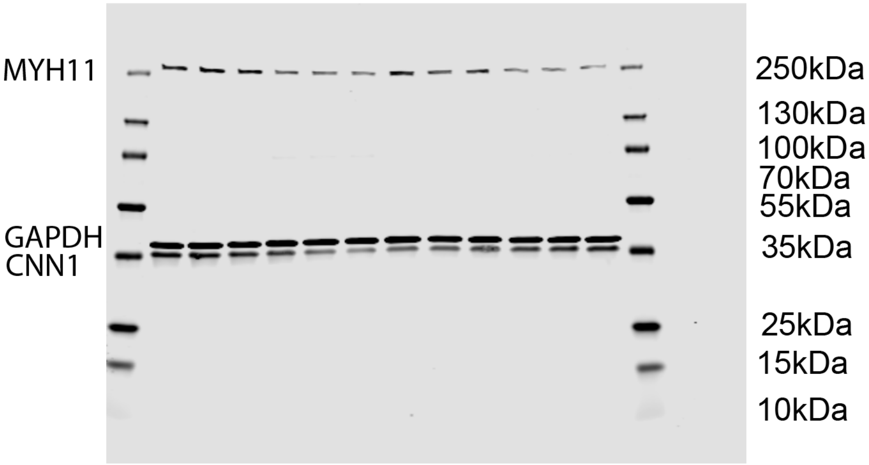


Full unedited blot for Supplemental Figure 3D

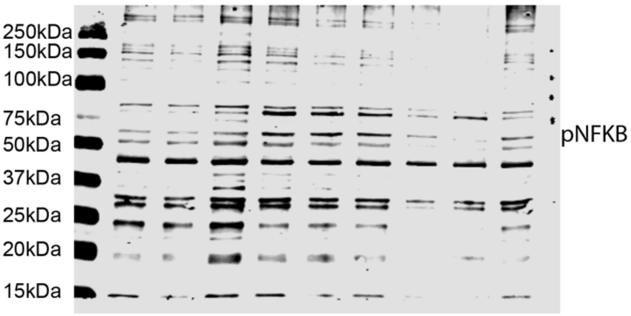


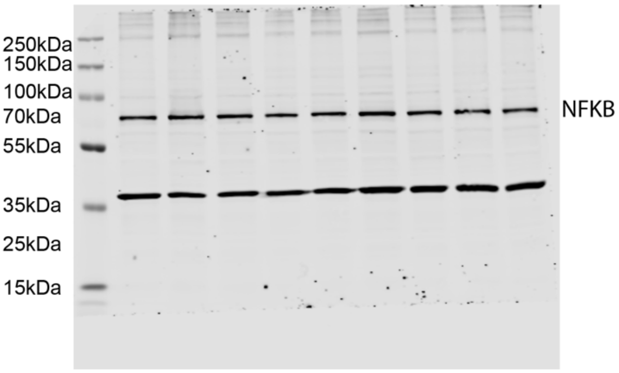


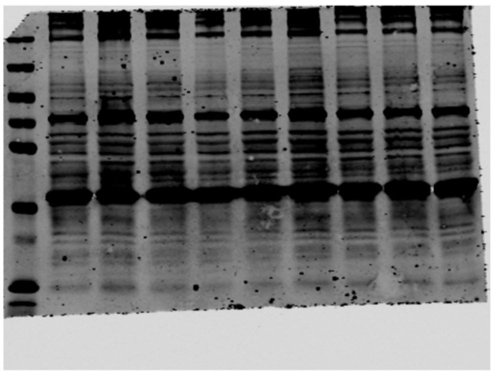

Full unedited blot for Supplemental Figure 3E

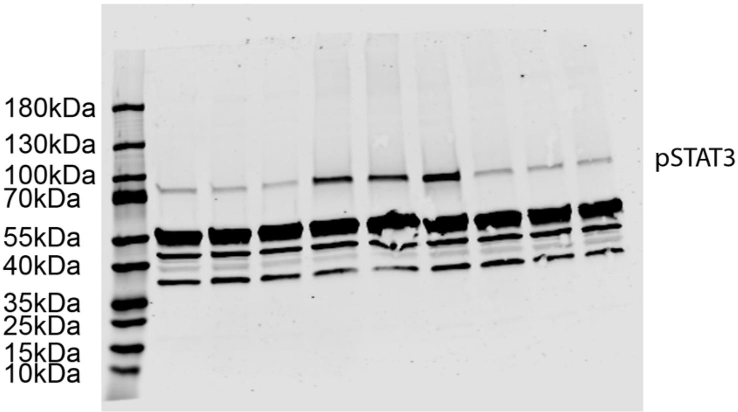


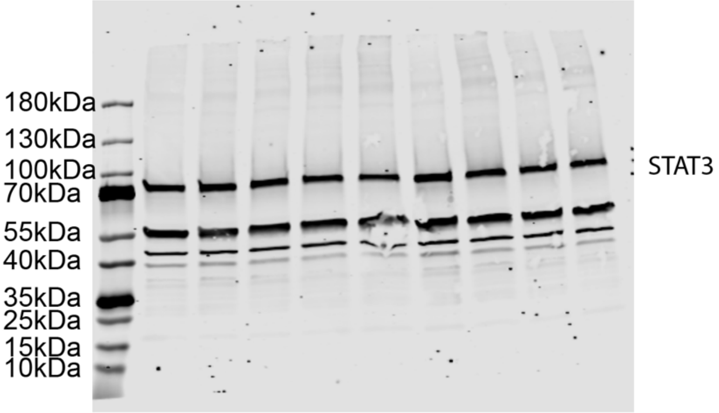


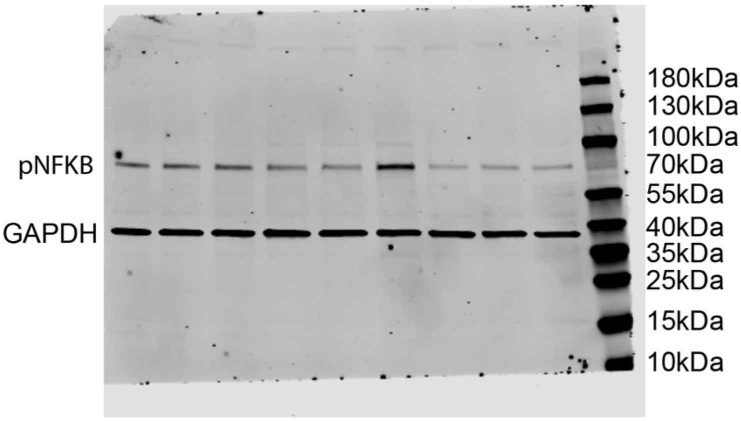


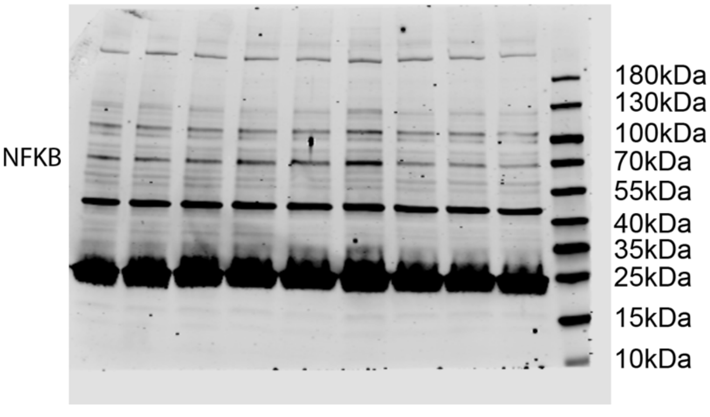

Full unedited blot for Supplemental Figure 3F

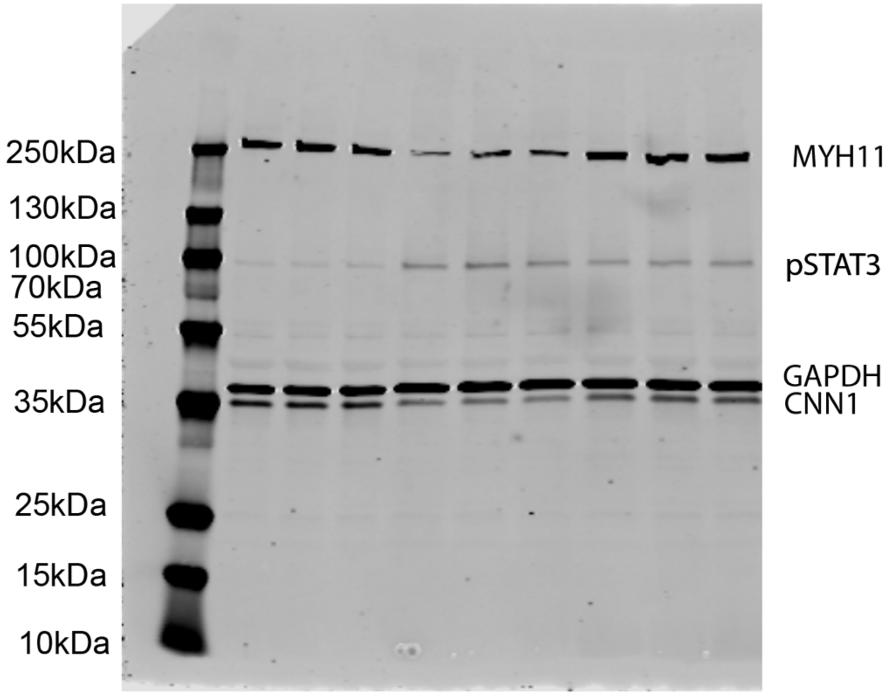


STAT3

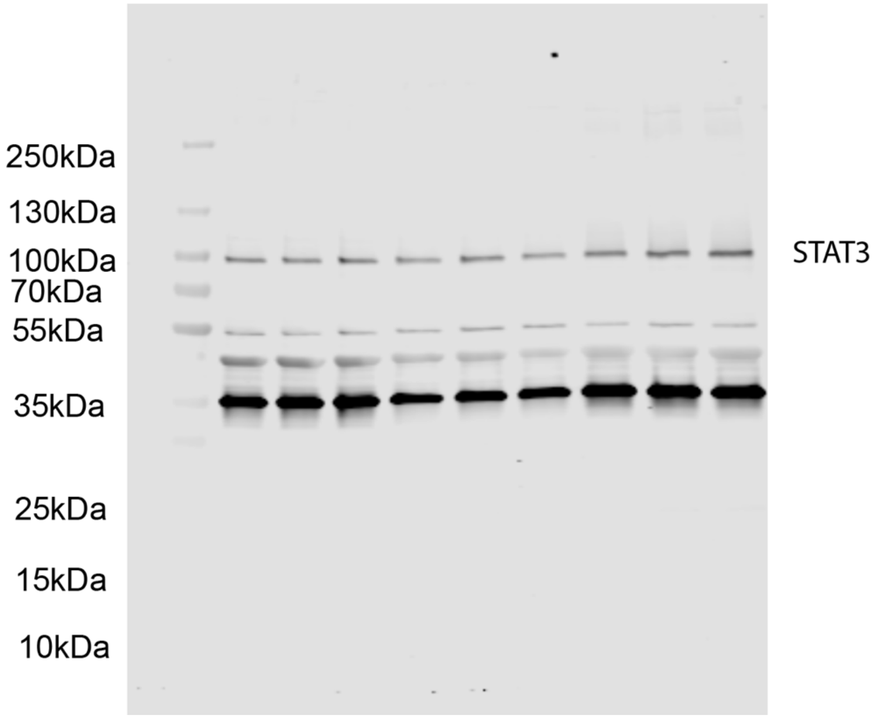

Supplement: Unedited blot and gel images [file jciinsight-11-200255-s231.pdf]
